# Transcortical approach surgery versus external ventricular drainage in treating intraventricular hemorrhage

**DOI:** 10.1097/MD.0000000000027443

**Published:** 2021-10-22

**Authors:** Jiahao Su, Yichun Xing, Sitao Liang, Qichang Lin, Huijiao Liu

**Affiliations:** a Neurosurgery Department, Zhongshan City People's Hospital, Zhongshan, China; b Department of Gynecology, Sun Yat-sen Memorial Hospital Affiliated of Sun Yat-sen University, Guangzhou, China; c Department of Critical Care Medicine, Zhongshan City People's Hospital, Zhongshan, China.

**Keywords:** extraventricular drainage, intraventricular hemorrhage, transcortical approach surgery

## Abstract

Intraventricular hemorrhage is a serious intracerebral hemorrhagic disease with high mortality and poor prognosis. This retrospective study designed to investigate the therapeutic effect of transcortical approach surgery versus extraventricular drainage (EVD) on patients with intraventricular hemorrhage.

Patients with intraventricular hemorrhage in Zhongshan City People's Hospital from January 01, 2014 to June 01, 2019 were retrospectively examined. They were divided into transcortical approach surgery groups and EVD groups to analyze the clinical characteristics and prognosis.

A total of 96 patients were enrolled in the study (24 in the transcortical approach surgery group and 72 in the EVD group). The efficiency of postoperative operation was 15/19 in the transcortical approach surgery group and 24/48 in the EVD group (*P* = .012). The Glasgow Outcome Scale was 3.63 ± 1.27 in the transcortical approach surgery group and 2.80 ± 1.87 in the EVD group (*P* = .049). The postoperative residual blood volume was 9.62 ± 3.64 mL in the transcortical approach surgery group and 33.60 ± 3.53 mL in the EVD group (*P* < .001). The incidence of hydrocephalus after the operation was 1/23 in the transcortical approach surgery group and 19/53 in the EVD group. The 30-day postoperative mortality was 16/56 in the EVD group and 1/23 in the transcortical approach surgery group. The transcortical approach surgery group was significantly better compared with the EVD group.

This study showed that the transcortical approach for ventricular hemorrhage compared with EVD improved the hematoma clearance rate, shortened catheterization time, reduced the incidence of postoperative hydrocephalus, decreased patient mortality, led to a better prognosis, and reduced complications of hydrocephalus.

## 1 Introduction

Ventricular hemorrhage is a serious central nervous system hemorrhagic disease. It can be caused by primary intraventricular hemorrhage or other kinds of cerebral hemorrhage into the ventricles, mostly in the thalamus, basal ganglia cerebral hemorrhage, or aneurysm rupture.^[1,2]^ A large amount of blood leaks into the ventricles and can be coagulated into blood clots, blocking the ventricular circulatory system, rapidly forming hydrocephalus, causing patients to stun, destroying nervous system function, and leading to extremely high mortality and disability.^[3,4]^ Traditional medical treatment cannot effectively relieve intracranial hypertension, and the treatment effect is poor. A combination of extraventricular drainage (EVD) and urokinase thrombolytic therapy is widely used.^[5–7]^ However, prolonged catheterization and injection often lead to severe intracranial infection, resulting in complications such as hydrocephalus. Also, the treatment effect is not ideal.^[7]^ At present, with the development of microsurgical surgery, especially the wide application of transcortial approach surgery technology, it has achieved good results by clearing the intraventricular hematoma by the transcortical approach surgery.^[8–10]^ However, the frontal keyhole surgery may cost more time and lead to more blood loss. The effect of frontal keyhole surgery on ventricular hemorrhage is still controversial.^[11]^ Therefore, this retrospective study was designed to investigate the efficacy of transsphenoidal hematoma evacuation versus EVD for treating intraventricular hemorrhage.

## 2 Methods

### 2.1 Patients and grouping

This was a retrospective cohort study approved by the Ethics committee of Zhongshan City People's Hospital. Patients with ventricular hemorrhage admitted to the Zhongshan City People's Hospital from January 01, 2014 to June 01 2019, were retrospectively examined. According to the surgical method, the patients were divided into transcortical approach surgery and EVD groups. A total of 96 patients were enrolled in the study (24 in the keyhole surgery group and 72 in the EVD group). The amount of bleeding was calculated using the 3D-Slicer Software (http://www.slicer.org). The basic information for inclusion in the 2 groups is shown in Table 1. No statistically significant difference was found between the 2 groups.

**Table 1:** Baseline clinical features for 96 IVH patients.

	Microsurgery group (24)	EVD group (72)	*P* value
Age (yr)	54.21 ± 9.81	52.75 ± 12.82	.61
Gender (M)	17 (0.78)	47 (0.65)	.62
GCS	9.05 ± 3.15	9.60 ± 3.66	.062
Volume (mL)	39.75 ± 13.57	36.11 ± 14.55	.28
Graeb score	7.33 ± 2.06	6.81 ± 2.27	.315

### 2.2 Surgical methods

**Figure 1:**
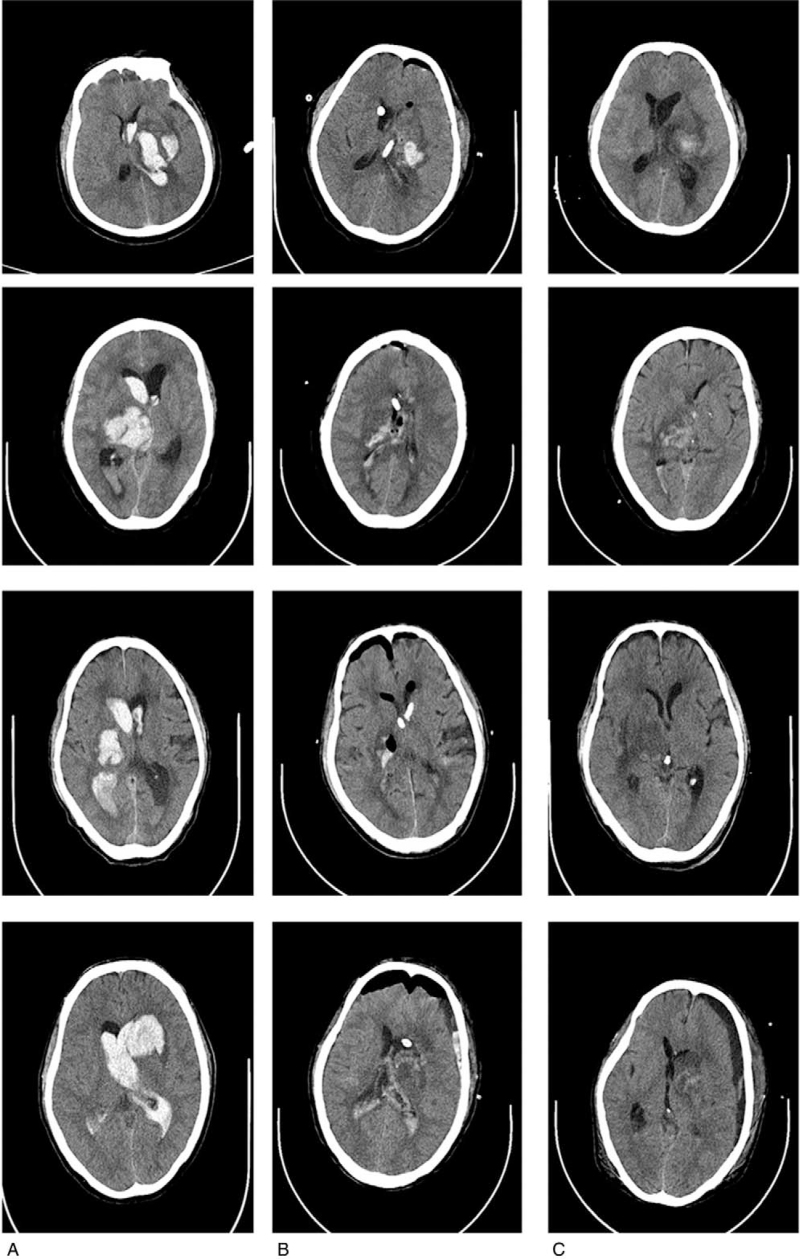
Pre- and postoperative CT scan images of severe IVH treated by transcortical approach surgery. (A) CT scan image at onset, with IVH and ventricular enlargement. (B) CT scan image 12 h after the transcortical approach surgery; the brain hematoma was mostly cleared. (C) CT scan images in the first week after the surgery. The hematoma was absorbed, with no significant edema and other injuries. CT, computed tomography, IVH = intraventricular hemorrhage.

The transcortical approach surgery: After successful anesthesia, the patient was placed in a supine position. The upper body was raised 15 degrees, the head was moved slightly to the side, the head frame was fixed to the head, and the head was routinely disinfected. A curved incision was made in the forehead. The length of the incision was about 8 cm. The whole layer was cut from the epidermis to the skull. The scalp flap was peeled off under the epithelium of the skull. The flap was turned to the skull base. The size of the bone window was 3.0 cm × 3.0 cm. The coronal suture was located on the posterior one-third of the bone window, the inner edge of the bone window was 1 cm from the midline, and the dura mater was suspended every 1.5 cm around the bone window. The X-shaped shearing of the dura mater was performed. A 2-cm incision was made into the longitudinal section of the frontal cortex. Entering the lateral ventricle, the intracranial blood clot was absorbed, the basal ganglia or thalamic hematoma was cleared, the hemorrhage point was electrocoagulated, and the intraventricular blood clot was continuously cleared. A No.14 tube was placed, and the microscope was removed. The incision was sutured intermittently, the cranial cavity was closed, the bone flap was repositioned, and then the incision was sutured in full thickness (Fig. 1 and Fig. 3).

Extracorporeal drainage: After successful anesthesia, the patient was placed in a supine position, and the head was conventionally disinfected using a field towel. The skin was cut first on the right side of the hairline (incision length about 3 cm), with 3 cm as the center line (puncture point) and parallel to the sagittal line. A hole was drilled into the skull, bipolar coagulation of the dura mater was performed, and the dura mater was cut using a sharp knife. Using a puncture needle, the ventricle was punctured to the midpoint of the double external auditory canal through the puncture point. The right external cerebral drainage was performed, and a ventricle drainage tube No. 14 was placed.

**Figure 2:**
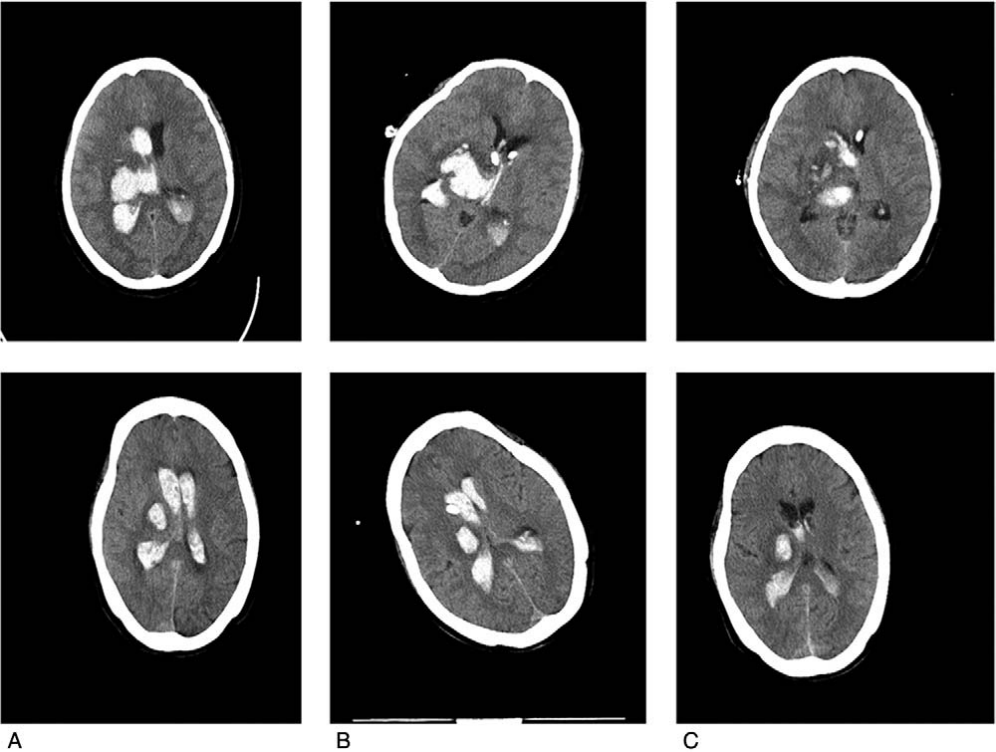
Pre- and postoperative CT scan images of severe IVH treated by EVD. (A) CT scan image at onset, with IVH and ventricular enlargement. (B) CT scan image 12 h after the EVD; most hematoma was not cleared. (C) CT scan images in the first week after the surgery. Most hematoma was absorbed, and the tube was still placed in the ventricle. CT, computed tomography, EVD = extraventricular drainage, IVH = intraventricular hemorrhage.

The head computed tomography was reviewed 12 hours after the operation, and the residual blood volume was reassessed (Fig. 2).

**Figure 3:**
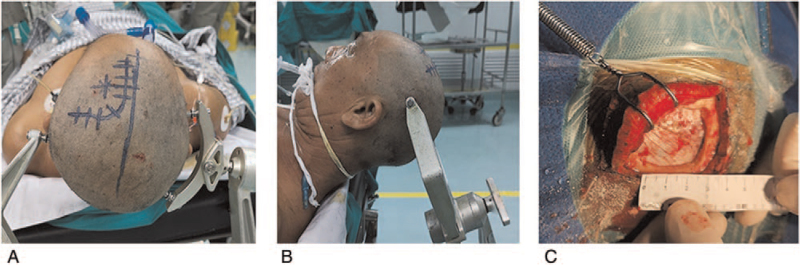
(A) Head in the coronal position; (B) the water-level view; (C) bone window size. The bone window size was about 3 cm × 3 cm.

### 2.3 Statistical analysis

The data were analyzed using the SPSS20.0 software. They were expressed as the mean and standard deviation or number and percentage. The rate comparisons were performed using the chi-square test and Fisher's exact test. The threshold for statistical significance was set at *P* = .05.

## 3 Results

### 3.1 Surgery-related results

The intraoperative conditions included intraoperative blood loss, operation time, postoperative residual blood volume, postoperative rebleeding, secondary surgery, and postoperative Graeb score. The intraoperative blood loss and operation time were significantly better in the EVD group than in the transcortical approach surgery. The postoperative residual blood volume, hematoma clear rate, and Graeb score in the transcortical approach surgery were superior to those in the EVD group. The difference was statistically significant (Table 2).

**Table 2:** Surgery-related results.

	Microsurgery group (24)	EVD group (72)	*P* value
Volume (mL)	9.62 ± 9.13	33.60 ± 15.28	<.001
Time (min)	164.83 ± 37.35	61.33 ± 17.49	<.001
Clearance rate	0.753 ± 0.247	0.06 ± 0.225	<.001
Rebleeding (Y/N)	4 (0.17)	11 (0.15)	.871
Reoperation (Y/N)	3 (0.125)	4 (0.056)	.257
Bleeding (mL)	110.83 ± 91.79	21.80 ± 23.05	<.001
Post Graeb score	3.17 ± 2.56	6.54 ± 2.15	<.001

### 3.2 Treatment effect

The Glasgow Coma Scale (GCS) score, Graeb scores, and Glasgow Outcome Scale (GOS) 6 months after the operation were analyzed. The improvement in the GCS score ≥ 3 was significant. No significant difference in the GCS\GOS was found between the 2 groups. The effective rate in the transcortical approach surgery was superior to that in the external drainage group, and the difference was statistically significant. On the first postoperative day, the head computed tomography was re-evaluated to calculate the residual blood volume in the brain and the contrast between the 2 groups. The amount of bleeding was less in the keyhole group than in the external drainage group. The difference was statistically significant. The postoperative Graeb score was less in the transcortical approach surgery than in the EVD group. The duration of hospitalization and postoperative catheterization time was less in the transcortical approach surgery than in the external drainage group. The difference was statistically significant (Table 3).

**Table 3:** Treatment effect for 96 patients with IVH.

	Microsurgery group (24)	EVD group (72)	*P* value
Post GCS	10.91 ± 3.95	9.44 ± 5.42	.22
GOS	3.63 ± 1.27	2.80 ± 1.87	.049
Tube time (d)	2.46 ± 1.64	5.67 ± 1.64	.000
Hospital stay (d)	28.71 ± 21.62	31.50 ± 25.97	.637
Effective rate (Y/N)	15 (0.625)	24 (0.33)	.012
Post mRS	3.50 ± 1.62	3.67 ± 2.06	.719

### 3.3 Postoperative complications

A total of 17 patients died 30 days after the surgery, including 16 in the external drainage group and 1 in the transcortical approach surgery. The postoperative complications included hydrocephalus, wound healing disorders, pneumonia, urinary tract infections, epilepsy, and gastrointestinal bleeding. One patient had the occlusion of the ventriculoperitoneal shunt and 19 had external drainage. The transcortical approach surgery was superior to the external drainage group. The difference was statistically significant. Postoperative pneumonia and intracranial infection in the transcortical approach surgery were superior to those in the external drainage group (Table 4).

**Table 4:** Adverse event.

	Microsurgery group (24)	EVD group (72)	*P* value
Pneumonia	14 (0.58)	42 (0.58)	1
Brain infection	1 (0.04)	15 (0.21)	.048
Cerebral infarction	1 (0.04)	3 (0.04)	1
Hydrocephalus	1 (0.04)	19 (0.26)	.020
Urine tract infection	5 (0.21)	17 (0.24)	.779
Healing disorder	5 (0.21)	9 (0.125)	.316
Epilepsy	1 (0.04)	1 (0.014)	.409
Death	1 (0.04)	16 (0.22)	.036
Gastrointestinal bleeding	4 (0.17)	15 (0.21)	.657

## 4 Discussion

Intraventricular hemorrhage (IVH) is a common and serious neurosurgical disease, which is mostly caused by spontaneous intracerebral hemorrhage.^[12]^ In particular, the prognosis of severe intraventricular hemorrhage is worse and the mortality rate is higher. At this time, most of the ventricular system is filled with blood or blood clots, resulting in the obstruction of cerebrospinal fluid circulation pathway and the formation of acute hydrocephalus. The traditional method is to place a disturbance external drainage tube, but due to the large volume of intraventricular hemorrhage, and the drainage tube is often blocked by blood clots, it is difficult to drain blood in time, resulting in poor treatment effect. We adopted minimally invasive surgery through frontal cortex approach, which cleared the blood of ventricle under direct vision, and achieved better prognosis than EVD alone.

In this study, patients with ventricular hemorrhage admitted to the hospital from January 01, 2014 to June 01, 2019 were retrospectively examined. The GCS score in January was ≥3 points higher than that before the surgery, which was effective for treatment. In this study, the keyhole group was better than the EVD group. The GOS score in the keyhole group was superior to that in the external drainage group 6 months after the surgery. The postoperative GCS score in the keyhole group was also superior to that in the EVD group, but the difference was not statistically significant. The results suggested that the keyhole surgery could improve the prognosis of some patients. The same results were also demonstrated in a study on neuroendoscopic intraventricular hematoma evacuation.^[1,11,13]^ Despite using different surgical techniques, the keyhole surgery was also superior to simple EVD surgery.

In terms of the 30-day postoperative mortality rate, the keyhole group was significantly better than the EVD group. The ventricular hemorrhage originated mainly from the cerebral hemorrhage in the thalamus and basal ganglia. In this study, 12 patients in the transcortial approach surgery group had thalamic hemorrhage, and 46 patients in the EVD group had thalamic or basal ganglia hemorrhage. The main cause of mortality due to thalamic hemorrhage is that the thalamus is close to the 3 ventricles and the brainstem; cerebral edema caused by thalamic hemorrhage can easily compress the brainstem and the third ventricle, leading to serious complications.^[14,15]^ EVD can only reduce the occurrence of hydrocephalus but also reduce the compression of surrounding structures. The transcortial approach surgery can clear a thalamic hematoma and reduce the secondary oppression while clearing an intraventricular hematoma, thus reducing mortality. The transcortial approach surgery group of patients who underwent ventriculoperitoneal shunt in postoperative hydrocephalus was also superior to the EVD group. It has been reported^[16,17]^ that intraventricular hemorrhage is an independent prognostic factor for IVH, and the key step in the treatment of intraventricular hemorrhage is to clear the intraventricular blood as soon as possible. Transcortical microsurgery could clear primary hematoma and intraventricular hematoma under direct vision, rapidly reduce intracranial pressure, and improve cerebral blood flow perfusion; in addition, it can solve the blockage of cerebrospinal fluid circulation and reverse ventricular dilatation. Hydrocephalus is easily caused by the decomposition products of blood in the ventricle.^[18]^ The intracerebroventricular hematoma was removed rapidly through frontal approach, thus the incidence of hydrocephalus was reduced. All these measures may reduce the need of VP shunt operation to some extent. Our results are similar to those reported by other researchers.^[1,19–21]^ The transcortical approach of microsurgery and endoscopic surgery significantly reduces permanent shunt dependency for IVH. The incidence of postoperative intracranial infection was also lower than that of the EVD group. Through analysis, we found that the transcortical approach of microsurgery could quickly clear the hematoma, reduce the indwelling time of drainage tube, so as to reduce the incidence of intracranial infection. This result is similar to Toyooka's report^[22]^ that early surgical intervention for intracerebral hemorrhage patients with IVH.

In terms of complications, the operation time in the transcortial approach surgery group was better than that in the EVD group. No significant difference was found in the rate of secondary bleeding after the surgery. Also, no significant differences were observed in postoperative complications, gastrointestinal bleeding, urinary tract infection, and wound healing disorders. Further, the hospital stay had no significant difference. The incidence of postoperative pneumonia was better in the transcortial approach surgery group than in the EVD group.

A large number of studies achieved good results using the keyhole endoscopic surgery to remove an intraventricular hematoma.^[11,20–22]^ This study used microscopic techniques to remove an intraventricular hematoma. Compared with the Ping SONG2018^[1]^ study, no significant difference was found between the 2 procedures in terms of hematoma clearance rate and effective rate. Microsurgical incisions and bone flaps were slightly larger compared with endoscopy. The duration of microsurgery was longer than that of endoscopic surgery. Endoscopic surgery provides a deeper intraventricular view. Microsurgery can avoid the fisheye effect while removing an intracerebral hematoma, and it is easier for neurosurgeons to master. Especially for the underdeveloped areas of neurosurgeons who do not have neuroendoscopy equipment, these techniques have greater operational significance (Table 5).

**Table 5:** Endoscopic surgery versus microsurgery in the treatment of IVH.

	Microsurgery group (24)	Endoscopic (18)	*P* value
Pro GOS	7.67 ± 3.24	9.27 ± 2.80	.099
Post GCS	10.83 ± 4.24	11.33 ± 3.65	.69
Death	1 (0.04)	1 (0.056)	.834
Clear rate	0.75 ± 0.25	0.71 ± 0.28	.587
GOS	3.62 ± 1.27	3.83 ± 1.15	.589

The major limitation of this study was its retrospective cohort design. Hence, offsets could not be avoided at the beginning of the study. Further, a small sample size made it impossible to conduct an in-depth analysis of the specific causes of bleeding. Patients did not have long-term follow-up, and hence long-term effects could not be evaluated.

This study found that the use of transcortial approach surgery to remove an intraventricular hematoma, compared with the combination of EVD and urokinase intraventricular injection, could achieve higher efficiency and reduce the mortality of patients after 30 days. It also reduced the incidence of postoperative intracranial infection and hydrocephalus. No significant difference was found in the effect of transforaminal surgery compared with neuroendoscopic surgery. It is important to treat intraventricular hemorrhage with transcortial approach surgery in underdeveloped areas lacking widely used neuroendoscopy techniques.

## Author contributions

**Investigation:** Jiahao Su, Yichun Xing, Sitao Liang, Qichang Lin, Huijiao Liu.

**Methodology:** Jiahao Su, Yichun Xing, Sitao Liang, Qichang Lin, Huijiao Liu.

**Validation:** Jiahao Su.

**Visualization:** Jiahao Su, Yichun Xing.

**Writing – original draft:** Jiahao Su, Yichun Xing, Sitao Liang, Qichang Lin, Huijiao Liu.

**Writing – review & editing:** Jiahao Su, Yichun Xing, Sitao Liang, Qichang Lin, Huijiao Liu.
